# Network analysis of the relationships among burnout, presenteeism, and social support in Chinese pediatric nurses

**DOI:** 10.3389/fpubh.2026.1835612

**Published:** 2026-06-25

**Authors:** Yan Tang, Deliang Kong, Jiajia Zhou, Yong Li, Shanwei Li, Chuan Pu

**Affiliations:** 1 School of Public Health, Chongqing Medical University, Chongqing, China; 2 Department of General Surgery and Trauma Surgery, Children's Hospital of Chongqing Medical University, National Clinical Research Center for Children and Adolescents' Health and Diseases, Ministry of Education Key Laboratory of Child Development and Disorders, Intelligent Application of Big Data in Pediatrics Engineering Research Center of Chongqing Education Commission of China, Chongqing, China; 3 School of Population and Health, Renmin University of China, Beijing, China

**Keywords:** burnout, network analysis, network estimation and visualization, node centrality, pediatric nurses, presenteeism, social support

## Abstract

**Background:**

Burnout and presenteeism are occupational health problems affecting professional stability in the nursing workforce and the quality of healthcare services. Social support is considered to be associated with burnout and presenteeism; however, the symptom-level association patterns among these three constructs remain unclear. This study systematically explored the structural associations and node characteristics among burnout, presenteeism, and social support based on a network analysis approach.

**Methods:**

A questionnaire survey was conducted among pediatric nurses in Chongqing, Southwest China, using a convenience sampling method, yielding 876 valid samples. A Gaussian graphical model (GGM) was constructed based on the Spearman correlation matrix, and the regularized partial correlation network was estimated using the EBIC-glasso algorithm. Network structure was computed using R software, and centrality indices, including strength, betweenness, closeness, and expected influence were derived. The accuracy of edge weight and the stability of centrality measures were assessed through 1,000 nonparametric bootstrap samples.

**Results:**

Presenteeism was positively correlated with burnout (r = 0.433), social support was negatively correlated with burnout (r = −0.361), and social support was negatively correlated with presenteeism (r = −0.179) (all p < 0.05). The strongest edge weight in the network structure was 0.72. Within the burnout module, D6 showed a strong connection with D5 and was associated with the depersonalization item E1, indicating that emotional exhaustion occupied a core position in the network. Centrality analysis demonstrated that E1 (Strength = 1.173), C11 (Strength = 1.122), and D6 (Strength = 1.112) were influential nodes. D1 and C3 showed the highest betweenness (Bet. = 97), playing bridging roles. Bootstrap results indicated narrow confidence intervals for edge weights, and the strength index maintained correlations >0.70 when 50% of the sample was retained, suggesting acceptable stability for strength-related findings in this sample.

**Conclusions:**

There were interactive relationships among burnout, presenteeism, and social support in pediatric nurses. Items related to emotional exhaustion were relevant nodes linking depersonalization and presenteeism, while family and emergency-related support resources played roles within the social support module. Network analysis identified relevant intervention nodes in occupational health and provides a reference for promoting occupational health

## Introduction

Burnout is a prolonged response to chronic emotional and interpersonal stressors on the job, and is defined by the three dimensions of exhaustion, cynicism, and inefficacy (1). It has become a critical public health concern in the global nursing field, exerting an impact on the physical and mental health of nursing professionals as well as on the stable operation of the healthcare service system. In the ICD-11, the World Health Organization explicitly defines burnout as an “occupational phenomenon,” emphasizing that it occurs in the work context and is associated with unsuccessful management of chronic workplace stress (2). Among nursing professionals, burnout is not only a problem of individual psychological depletion but may also be associated with risks through emotional contagion, impaired team collaboration, and decreased quality of nursing care (3). A network analysis study of Chinese clinical nurses showed that burnout, anxiety, and depression were highly correlated among nursing professionals; depersonalization (DP) was associated with adverse work outcomes, whereas emotional exhaustion was a predictor of poor quality of life (4). A systematic review and meta-analysis encompassing 45,539 nurses across 49 countries reported that the pooled prevalence of burnout symptoms among nurses worldwide was 11.23% (95% CI: 8.83–13.63). Among specialties, pediatric nurses had a relatively higher prevalence of burnout symptoms, suggesting that the high workload and high emotional labor in pediatric nursing settings may be distinctive (5). Compared with general wards, pediatric nursing, as a specialized field of medical services, exposes its practitioners to composite stressors distinct from other departments, higher-density communication with family members, and more frequent unexpected emergencies. Previous studies in China have also indicated significant associations among occupational stress, coping styles, and mental health in pediatric nurses (6).

Accompanying occupational burnout, and similarly concealed yet more challenging in management, is presenteeism, also referred to as “working while ill” or “impaired health-related productivity.” It generally refers to the phenomenon in which individuals continue to attend work despite being in a state of physical or psychological discomfort. Its core harm lies in reduced productivity and an increased risk of potential errors risk under conditions of impaired health (7). Groundbreaking work by Aronsson et al. provided an empirical description of working while ill, highlighting its complex associations with individual health, job irreplaceability, and absenteeism behavior (8). Among nursing populations, presenteeism not only affects the quality of nursing procedures and communication efficiency, but may also be associated with patient safety risks related to fatigue, decreased attention, and increased errors. Moreover, presenteeism is often associated with burnout. A study of Chinese nurses found that emotional labor was associated with presenteeism, suggesting that the burden of emotional labor may be associated with experiences of fatigue and “low efficiency while on duty” (9). Another study also confirmed the association between burnout and presenteeism (10). Results from a multiple mediation model showed that sickness presenteeism and job burnout were associated in the relationship between perceived stress and turnover intention, suggesting that illness or presenteeism may be an identifiable signal prior to workforce loss (11).

Social support, as a core construct in the field of positive psychology, emphasizes the role of interpersonal relationship networks in individual mental health. It refers to the emotional and material support that individuals receive from others when facing pressures or challenges, generating feelings of care and attention are generated in the interaction between the individuals (12). In the above mechanism chain, the associations of social support with occupational stress, health outcomes, burnout, and presenteeism have also been confirmed by related studies. It can reduce perceived stress through emotional support, informational support, and instrumental support, and can enhance work engagement and occupational efficacy by strengthening sense of belonging and sense of control (13). In a sample of hospital nurses in Germany, Kowalski et al. found that the higher the level of hospital social capital, the lower the degree of nurses' emotional exhaustion, emphasizing the importance of social capital and organizational development in hospital management (14). Evidence from systematic reviews indicates that social support is associated with nurse burnout and is considered an element that should be prioritized in intervention design (15). In addition, in the Chinese nurse health cohort (TARGET), presenteeism, burnout, and social support were jointly associated with health-related productivity loss, suggesting that the governance of presenteeism should go beyond “health education” and needs to be advanced in coordination with organizational support, work recovery, and the construction of occupational psychological resources (7). Furthermore, social support is also systematically associated with turnover intention. A systematic review and meta-analysis showed that social support is related to turnover intention among clinical nurses; work environment factors, including social support from supervisors and colleagues, can enhance subordinates' confidence in career development and the intimacy between supervisors and subordinates (16).

The above studies indicate pairwise or multivariable associations among social support, occupational burnout, and Presenteeism. The present study constructed a network analysis incorporating these three constructs mainly based on the following limitations in current research and considerations. First, previous studies have demonstrated associations among some of these variables (10, 11, 14–16), but no study has directly and specifically examined the relationships among all three constructs. This study will describe the influence relationships and associated nodes among the three constructs, thereby promoting understanding of the association mechanisms related to occupational burnout and Presenteeism. Second, traditional correlational studies have typically used total scores or dimension scores as the unit of analysis (17, 18), and linear regression, correlation analysis, or latent variable models mainly reflect overall effects and general associations. These approaches do not identify the associations and coupling mechanisms between different items and across constructs, making it difficult to reveal the connections, bridge nodes, and coupling patterns among specific items (19–21). Third, pediatric nurses are chronically exposed to stressful contexts such as high-intensity emotional labor, heavy workload, and responses to emergencies (6, 22). Therefore, occupational burnout, sickness-related work behavior, and support resources in this population may form an association structure with specialty-specific characteristics, making it necessary to characterize these relationships among pediatric nurses. Fourth, these three constructs are functionally connected within the work context and respectively represent psychological exhaustion, sickness-related work behavior, and supportive resources; therefore, they can be examined within the same system to investigate their interactions (9, 10, 23, 24).

In terms of the methodological principles of network analysis, as an emerging quantitative research method, it conceptualizes psychosocial structures as a system of interacting nodes and uses Gaussian graphical models (GGM) and regularized partial correlation networks to visually present the strength and pathways of associations among variables, thereby providing an effective research tool for addressing occupational health problems (25). This method has been effectively applied in post-traumatic stress disorder (26), anxiety–depression comorbidity (27), and nurse burnout and related occupational psychological structures (28). However, among pediatric nurses, studies that incorporate burnout, presenteeism, and social support into the same network system and conduct item-level analyses remain relatively limited. Based on the above research foundation and research gaps, this study used item-level network analysis modeling to identify the association structure and relevant nodes among the three constructs and proposed the following research hypotheses. First, occupational burnout, presenteeism, and social support among pediatric nurses are interrelated and can form an interconnected network structure. Second, items related to presenteeism and social support may form connections with items related to occupational burnout and may form clearly connected nodes in the network, thereby identifying local association pathways and key nodes. Therefore, based on the Gaussian graphical model framework, this study aimed to construct an item-level regularized partial correlation network of burnout, presenteeism, and social support among Chinese pediatric nurses, to identify the overall network structure and associated nodes among the three constructs, and to clarify the connections among nodes related to psychological exhaustion, impaired work functioning, and supportive resources, thereby providing a basis for targeted occupational health interventions for pediatric nurses.

## Methods

### Study design and participants

This study adopted a cross-sectional design using convenience sampling. In Chongqing, Southwest China, according to the ratio of the number of healthcare institutions among the three major urban agglomerations in Chongqing reported in the local health statistical yearbook (metropolitan urban area: northeast Chongqing urban agglomeration: southeast Chongqing urban agglomeration = 14:7:2), healthcare institutions were selected within each region using convenience sampling for the survey. Ultimately, questionnaire surveys were conducted in 32 hospitals, and the surveyed institutions in each region generally met the planned sampling ratio. The institution type was mainly public hospitals, with 29 public hospitals and a total of 3 mixed-ownership and private medical institutions. Hospital types included general hospitals, pediatric specialty hospitals, and maternal and child healthcare hospitals, and hospital tiers included secondary and below, tertiary, and other levels. The study participants covered different hospital types and tiers, which could, to some extent, reflect the occupational characteristics and work contexts of pediatric nurses in Chongqing. A questionnaire survey was conducted among pediatric nurses selected through convenience sampling in Chongqing, According to Kendell's sample size calculation method (29), the required sample size should be 5–10 times the number of variables. A total of 54 variables were analyzed in this study. Considering a 20% invalid response rate, the required sample size was estimated to be 324–648 participants. The inclusion criteria were as follows: (I) possession of a registered nurse license of the People's Republic of China and at least 1 year of clinical nursing experience; (II) engagement in pediatric nursing in pediatric specialty hospitals or general hospitals within Chongqing; and (III) provision of informed consent and voluntary participation in this study. The exclusion criteria were as follows: (I) nursing interns/residents and registered nurses not engaged in pediatric nursing;(II) a history of psychiatric or psychological disorders; and (III) those who did not participate in the survey due to leave, training, or other reasons. A total of 951 questionnaires were initially collected. After screening and excluding those who did not meet the inclusion criteria, as well as removing questionnaires with completion times not meeting the required duration (< 150 s), low response quality, logical errors, or incomplete information, 75 questionnaires were excluded. Ultimately, 876 valid questionnaires were included in the analysis, yielding an effective response rate of 92.11%.

### Data collection

From September to November 2025, a survey was conducted among pediatric nurses using an online electronic questionnaire. Prior to data collection, all research staff and data collectors underwent comprehensive training on ethical principles and standardized procedures for questionnaire administration. This ensured a consistent and ethically sound approach across all stages of the study. The online electronic questionnaire and its corresponding QR code were distributed to them, and the survey methods, implementation requirements, and precautions were clarified to ensure the standardization and consistency of the investigation. After the data collectors understood the survey requirements and completed the training, during the on-site investigation phase, the data collectors distributed the questionnaire and its QR code to nurses using convenience sampling, who voluntarily participated and independently completed the questionnaire. At the beginning of each questionnaire, the study purpose, precautions, principles of information confidentiality, potential risks, and the significance of participation were clearly stated. This ensured that all participants voluntarily took part in the survey on the basis of a full understanding of the relevant information and potential risks. It was also explicitly stated that all data would be processed anonymously and that participants could withdraw from the study at any time without any penalty. Whether before or during the survey, if a nurse chose not to participate or not to continue participation, no data would be collected and the survey process would be terminated immediately. To improve the accuracy of responses and prevent duplicate submissions, the electronic questionnaire was set to allow only one submission per IP address. After the completion of data collection, the researchers downloaded the data from the questionnaire platform for subsequent analysis.

## Measurements

### Sociodemographic information

Sociodemographic information was collected via self-administered questionnaires. These baseline characteristics encompassed demographic variables such as gender, age, education level, marital status, parental status, and monthly income, as well as work-related factors including employment type, years of professional experience, job position, professional title, institution type, institutional tier, and department of employment.

### Burnout

Burnout was assessed using the internationally recognized MBI-GS (Maslach Burnout Inventory–General Survey) (30). The scale was adapted into Chinese by Li Chaoping et al. (31). It comprises 15 items and includes three dimensions: emotional exhaustion (6 items), depersonalization (3 items), and reduced professional efficacy (6 items). The scale uses a 7-point Likert scoring method ranging from 0 to 6, with higher scores indicating more severe burnout. The scale has demonstrated strong reliability and validity, with a Cronbach's α coefficient of 0.825 in prior validation studies. In this study, the internal consistency coefficients for the three subscales were 0.88, 0.83, and 0.82, respectively—each exceeding the recommended threshold of 0.70, thus confirming good reliability. The overall Cronbach's α coefficient for the scale in this study was 0.894, further supporting its internal consistency.

### Presenteeism scale

Presenteeism was assessed using the Stanford Presenteeism Scale–Short Form (32). The scale consists of 6 items and includes two dimensions: work limitations and work energy. A 5-point Likert scoring method was adopted. Items 5 and 6 were reverse-scored. The total score ranges from 6 to 30, with higher scores indicating more severe presenteeism behavior caused by health problems. The Cronbach's α coefficient of the scale was 0.862. In this study, the Cronbach's α coefficient was 0.835.

### Social support scale

Social support was assessed using the Chinese version of the Social Support Rating Scale (SSRS) (33), which is widely used to measure types of social support and their utilization. The scale consists of 14 items across three dimensions: objective support (3 items), subjective support (8 items), and utilization of social support (3 items). Within the subjective support dimension, the sources of support and care received from family members are divided into five categories: spouse (partner), parents, children, siblings, and other members. The total SSRS score is the sum of the scores of all dimensions. The total score ranges from 12 to 66, with higher scores indicating higher levels of social support. The Cronbach's α coefficient of the scale was 0.896. In this study, the Cronbach's α coefficient was 0.780.

### Statistical analysis

Descriptive statistical analyses were conducted using Excel 2019 and SPSS 21.0. Categorical variables were described using frequencies and percentages, whereas continuous variables were expressed as means and standard deviations. The procedures for network analysis followed the standardized guidelines published by Epskamp et al. (34). The analytical components included network estimation, network visualization, estimation of centrality indices, and assessment of network accuracy and stability.

### Network estimation and visualization techniques

Network analysis was conducted using R software (version 4.3.2). Gaussian graphical models (GGMs) were used to construct these symptom networks, and the graphical least absolute shrinkage and selection operator (glasso) was applied to build a regularized partial correlation network. Specifically, the “glasso” package (version 1.11) and the “qgraph” package (version 1.9.8) were used to construct and visualize the weighted network among nurse burnout, presenteeism, and social support. In these networks, each “node” represents a manifestation of nurse burnout, presenteeism, or social support, and each “edge” represents the partial correlation coefficient between pairs of nodes. Spearman correlation coefficients were used to calculate partial correlations while controlling for the influence of all other nodes. To reduce spurious correlations, the least absolute shrinkage and selection operator (LASSO) and the extended Bayesian information criterion (EBIC) were used to obtain a sparse regularized network. Network visualization was implemented using the Fruchterman–Reingold algorithm, in which nodes with more connections are positioned closer to the center of the network. The thickness of edges represents the strength of associations between nodes, and different types of symptoms are distinguished by different colors to more clearly present various symptom clusters.

### Node centrality

Node centrality is an index used to identify relevant features from a mechanistic perspective. Four centrality metrics were applied: strength centrality, betweenness centrality, closeness centrality, and expected influence. Higher centrality indices indicate that a node plays a more important role at the mechanistic level. Strength centrality refers to the sum of the edge weights connected to a given node. Betweenness centrality reflects the frequency with which a node appears on the shortest paths between all pairs of nodes, thereby representing its role and influence in connecting other nodes. Closeness centrality indicates the distance between a node and other nodes and is calculated as the reciprocal of the sum of the shortest path distances from that node to all other nodes. Expected influence quantifies the influence of a node within the entire network by calculating the expected impact of that node on other nodes.

### Assessment of network stability

To evaluate the accuracy and stability of these results, a bootstrap procedure based on the “bootnet” package (version 1.5.6) was applied. Using 1,000 nonparametric bootstrap samples, the network model was repeatedly estimated, and 95% confidence intervals of edge weights were calculated to assess the stability of edges. Node stability was evaluated by gradually removing certain samples during the bootstrap process to calculate the correlation stability coefficient. These coefficients represent the probability that, after removing a certain proportion of the sample, the parameter correlation between the reconstructed network and the original network exceeds 0.7. The minimum threshold was set at 0.25; when this probability exceeded 0.5, the corresponding centrality estimate was considered to show acceptable stability.

## Results

## Sociodemographic characteristics and correlation analysis

Among the nurses included in this study, females accounted for the vast majority (97.95%), and the age group of 31–40 years and above accounted for the highest proportion (55.25%). In terms of educational background, most nurses held a bachelor's degree (87.90%). Regarding years of work experience, 62.33% of nurses had worked for 5–15 years. In terms of professional titles, the title of nurse-in-charge was the most common (49.89%), while 87.88% of nurses held no administrative position. With respect to marital status, the majority of nurses were married (82.76%) and had children (78.65%), and 86.67% reported a monthly income not exceeding 10,000 CNY. Regarding employment type, most nurses were contract nurses (76.03%). Nurses working in tertiary hospitals accounted for 76.40% of the sample. In terms of departmental distribution, pediatric internal medicine nurses constituted the largest proportion (44.78%).Spearman correlation analysis was conducted for the three variables of presenteeism, social support, and burnout. The results showed that presenteeism was significantly positively correlated with burnout, whereas social support was significantly negatively correlated with presenteeism. The detailed results are shown in Table 1.

**Table 1:** Correlation analysis among presenteeism, social support and job burnout.

Items	Presenteeism	Social support
Presenteeism	1.00	
Social support	−0.179^a^	
Job burnout	0.433^a^	−0.361^a^

^a^*p* < 0.05.

## Network structure

**Figure 1:**
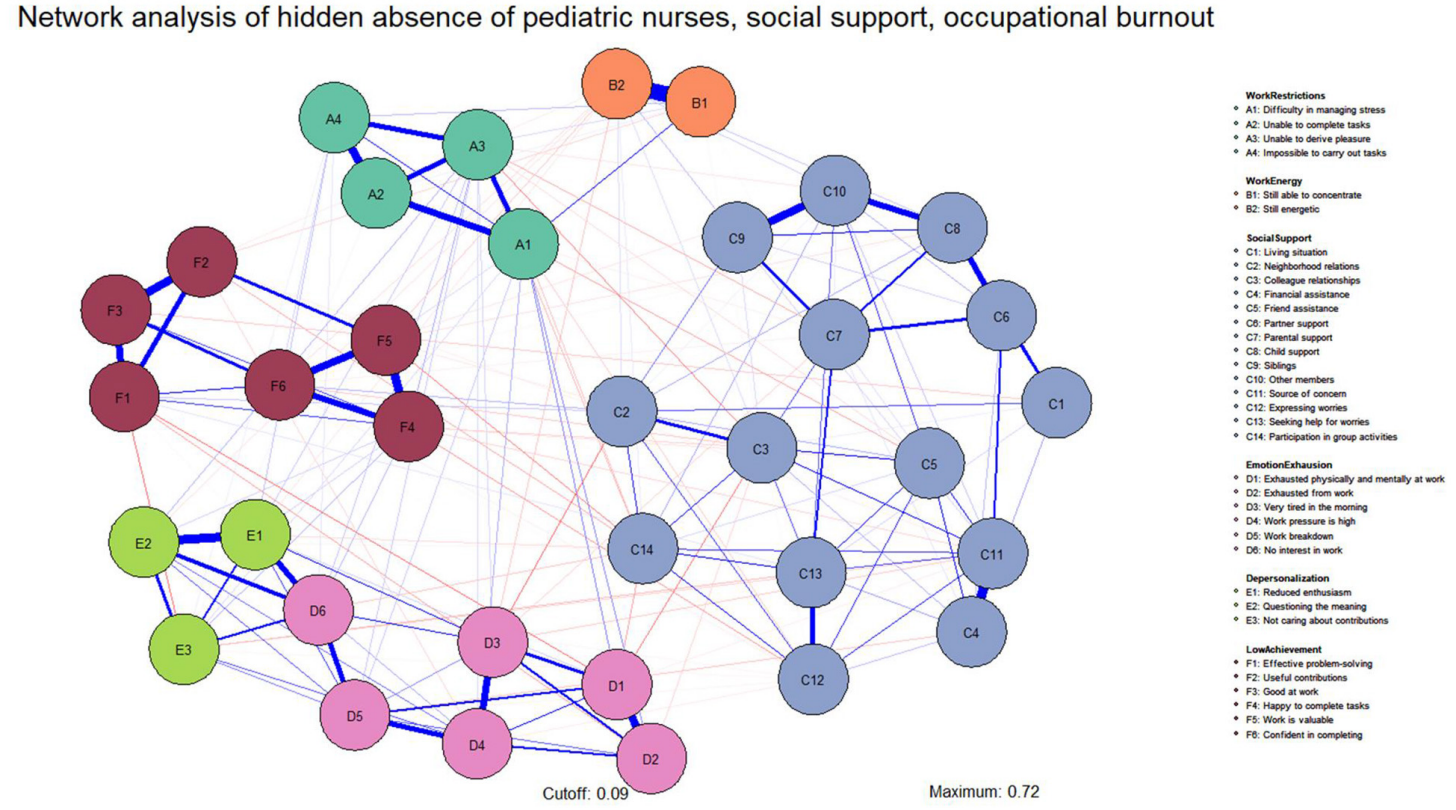
Network analysis of presenteeism, social support, and burnout among pediatric nurses. Nodes labeled **A** and **B** represent presenteeism (teal nodes: work restriction; orange nodes: work effort). Nodes labeled C represent social support (blue nodes). Nodes labeled D, E, and F represent burnout (pink nodes: emotional exhaustion; green nodes: depersonalization; red nodes: reduced personal accomplishment). Blue edges represent positive partial correlations, while red edges represent negative partial correlations. Shorter distances between nodes and thicker edges indicate stronger associations between items.

Figure 1 presents the network model of burnout, presenteeism, and social support among pediatric nurses. The strongest edge weight in the network analysis reached 0.72. Within presenteeism, in the work limitations dimension, node A3 (because of health problems, unable to obtain pleasure from work) showed the most significant connections with A2 (because of health problems, unable to complete difficult work tasks) and A4 (because of health problems, feeling that it is impossible to carry out certain work tasks), forming an associated subcluster of presenteeism, reflecting that presenteeism among pediatric nurses is manifested in reduced work-related pleasure and difficulty in completing work tasks. In the work energy dimension, the blue edge between B1 (despite health problems, still able to concentrate on completing work) and B2 (despite health problems, still feeling energetic enough to complete all work) was the thickest and shortest, indicating a relatively strong association between these two work-energy items.

Within burnout, node D6 (I have become more callous toward work since I took this job) had the strongest connection with D5 (I feel burned out from my work) within the same dimension and was also associated with E1 (I have become less enthusiastic about my work) and E2 (I doubt the significance of my work) in the depersonalization dimension.

In the reduced personal accomplishment dimension, node F4 (I feel very happy when I accomplish some tasks of my job) showed the most significant connections with F5 (I have accomplished many worthwhile things in this job) and F3 (In my opinion, I am good at my job), forming an associated subcluster within the reduced personal accomplishment dimension, reflecting that these symptoms highly co-occur among pediatric nurses. In the depersonalization dimension, node E1 (I have become less enthusiastic about my work) showed the connections with D6 (I have become more callous toward work since I took this job) and D5 (I feel burned out from my work) in the emotional exhaustion module. Multiple blue edges were observed between emotional exhaustion and depersonalization, indicating relatively close associations between emotional exhaustion and depersonalization items.

Within the social support dimension, node C6 (Support from spouse/partner) showed the strongest connections with C9 (Support from siblings) and C11 (Support from other members), suggesting that relatively strong associations among several family-related social-support items within the social-support module.

## Accuracy and Stability Analysis

**Figure 2:**
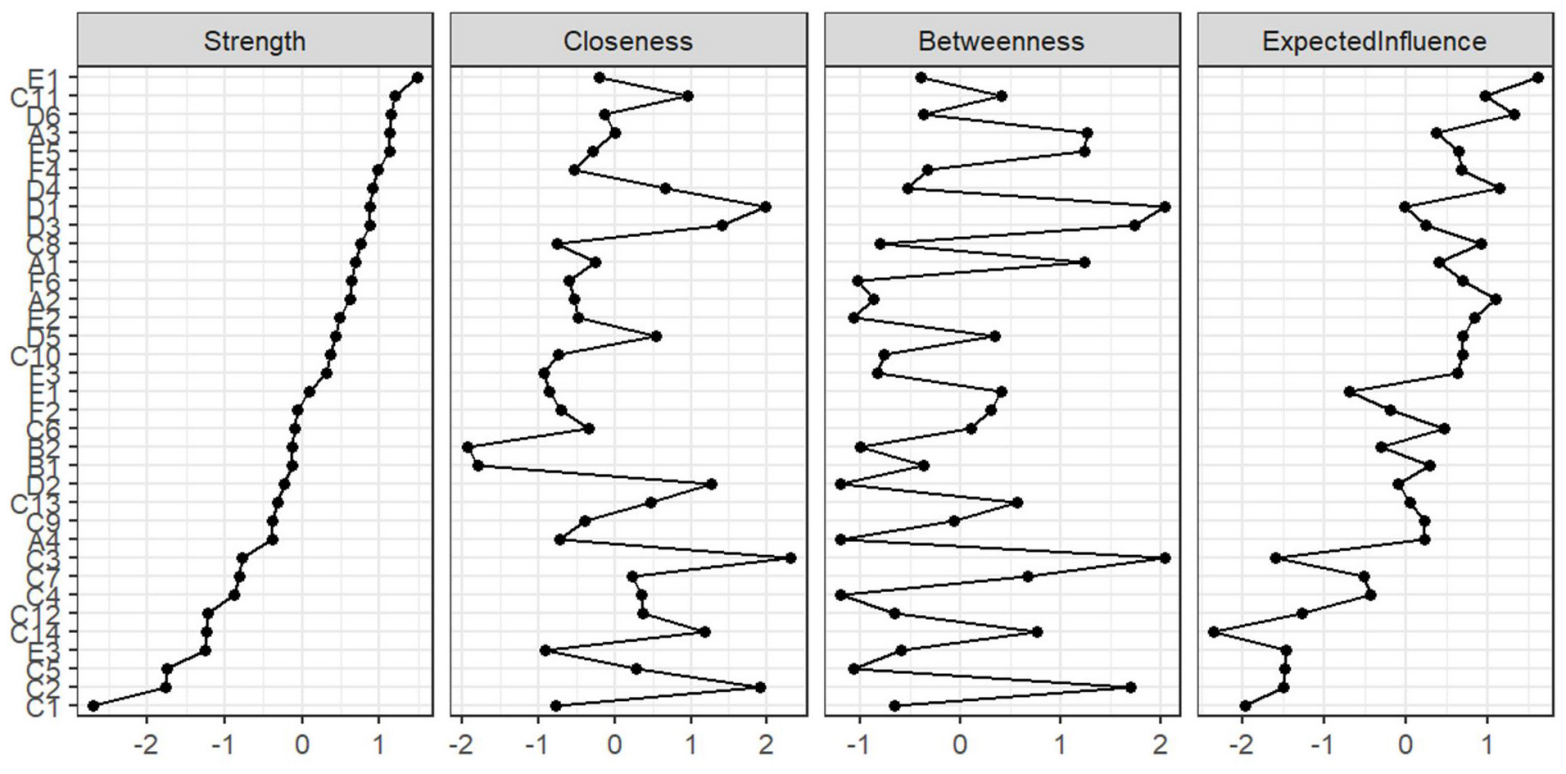
Comparison of network centrality measures across different indicators. Centrality indices, including strength, closeness, betweenness and expected-influence are plotted to show the relative importance of each indicator within the network structure.

The analysis of betweenness (Bet), closeness (Clo), and strength (Str) for each node in the model indicated differences in the topological positions and influence of different nodes within the symptom cluster network (Figure 2). The accuracy indices shown Figure 2 indicated that strength-related indices were more interpretable in this network, whereas betweenness showed relatively weaker performance. From the perspective of betweenness, nodes D1 and C3 had the highest betweenness values (Bet = 97), indicating that they served as hubs in the connections of the symptom network. This was followed by node D3 (Bet = 88), which also showed transmission capacity. In contrast, nodes A4, C4, and D2 had betweenness values of 0, indicating that these nodes existed only as peripheral nodes in the network in the association pathways among symptoms.

At the level of closeness, smaller closeness values indicate a more central topological position in the network. In this study, node B2 had the lowest closeness (Clo = 0.0007136329), with a corresponding strength value of 0.8898622, reflecting a relatively strong level of network connectivity. Node D1 had the highest closeness (Clo = 0.0011152711), indicating a relatively peripheral position in the network. The closeness values of the remaining nodes ranged from 0.0007281826 to 0.0010559438.

The strength index reflects the degree of association between a node and other nodes. Node E1 had the highest strength value (Str = 1.1733865), with a corresponding betweenness of 24 and closeness of 0.0008905131, collectively demonstrating its prominent influence within the network. This was followed by node C11 (Str = 1.1221620) and node D6 (Str = 1.1123135), both of which showed relatively strong node association strength, suggesting that these three nodes play key roles in the associations among burnout, presenteeism, and social support. In contrast, node C1 had the lowest strength value (Str = 0.4366615), with a betweenness of only 4, indicating relatively weak connectivity and influence within the network. Additionally, nodes C2 (Str = 0.6014712), C5 (Str = 0.6032752), C12 (Str = 0.6950045), C14 (Str = 0.6943813), and E3 (Str = 0.6904069) all had strength values below 0.7, indicating relatively limited contributions to the symptom cluster network.

Overall, D6, E1, and C11 were among the more influential core nodes in the symptom cluster network of this study. By connecting different symptom modules and association pathways, they played roles in the occurrence and development of burnout, presenteeism, and social support. In contrast, nodes C1, C2, C5, and C14 exhibited relatively limited network roles. Detailed centrality indices for each node are presented in Table 2.

**Table 2:** Centrality measures of presenteeism, social support, and job burnout indicators.

Items	Wording of the scale items	Betweenness	Closeness	Strength
Presenteeism				
A1	Work stresses much harder to handle because of health problems	73	0.0008861291	1.0315146
A2	Because of health problems, unable to complete difficult work tasks	10	0.0008571778	1.0216367
A3	Because of health problems, unable to obtain pleasure from work	74	0.0009122074	1.1113642
A4	Because of health problems, feeling that it is impossible to carry out certain work tasks	0	0.0008386749	0.8440376
B1	Despite health problems, still able to concentrate on completing work	25	0.0007281826	0.8874759
B2	Despite health problems, still feeling energetic enough to complete all work	6	0.0007136329	0.8898622
Social support				
C1	Living arrangement over the past year	16	0.0008322911	0.4366615
C2	Relationship with neighbors	87	0.0011075967	0.6014712
C3	Relationship with colleagues	97	0.0011487510	0.7758212
C4	Total sources of financial or practical support received during past emergencies	0	0.0009480840	0.7555087
C5	Sources of comfort and care during past emergencies: support from friends	4	0.0009417149	0.6032752
C6	Support from spouse/partner	39	0.0008778745	0.8938369
C7	Support from parents	56	0.0009355051	0.7681510
C8	Support from children	12	0.0008336980	1.0436512
C9	Support from siblings	34	0.0008722176	0.8446242
C10	Support from relatives/collateral family members	13	0.0008366656	0.9750786
C11	Support from other members	48	0.0010099148	1.1221620
C12	Ways of venting troubles	16	0.0009499487	0.6950045
C13	Ways of seeking help when troubled	53	0.0009600638	0.8561825
C14	Participation in group activities	59	0.0010326779	0.6943813
Burnout				
D1	I feel emotionally drained from my work	97	0.0011152711	1.0653903
D2	I feel used up at the end of the day	0	0.0010423906	0.8711874
D3	I feel tired when I get up in the morning and have to face another day at work	88	0.0010559438	1.0645347
D4	Working with people all day is a real strain for me	20	0.0009806539	1.0710674
D5	I feel burned out from my work	46	0.0009672615	0.9887518
D6	I have become more callous toward work since I took this job	25	0.0008983360	1.1123135
E1	I have become less enthusiastic about my work	24	0.0008905131	1.1733865
E2	I doubt the significance of my work	4	0.0008629662	0.9978461
E3	I have become more and more indifferent in the contribution of my job	18	0.0008184545	0.6904069
F1	I deal effectively with the problems arising in work	48	0.0008247340	0.9288494
F2	I feel that I am contributing to the hospital	45	0.0008390648	0.9017931
F3	In my opinion, I am good at my job	11	0.0008174342	0.9678645
F4	I feel very happy when I accomplish some tasks of my job	26	0.0008580715	1.0840216
F5	I have accomplished many worthwhile things in this job	73	0.0008828863	1.1098886
F6	I am confident that I can accomplish all tasks effectively	5	0.0008499508	1.0247535

**Figure 3:**
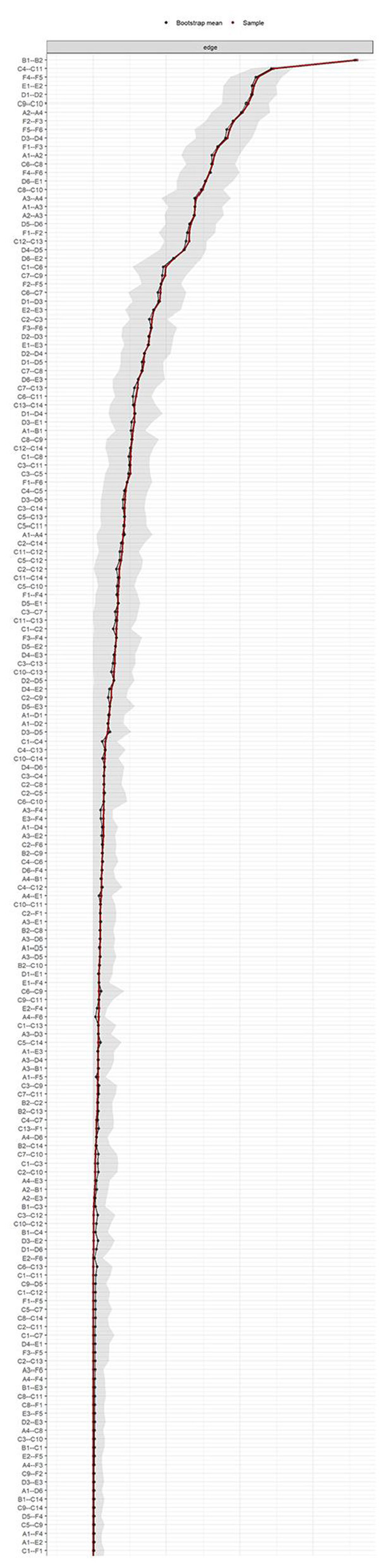
Bootstrap mean and sample values of network edges in presenteeism, social support and job burnout analysis. Mean bootstrap values represented by black nodes and original sample values represented by red line for each edge in the network comprising presenteeism, social support, and job burnout. Edges are displayed along the *x*-axis.

As shown in Figure 3, the accuracy results indicated that several edge weights among burnout, presenteeism, and social support were estimated with relatively narrow confidence intervals, suggesting acceptable precision for the more stable edges in the current network.

**Figure 4:**
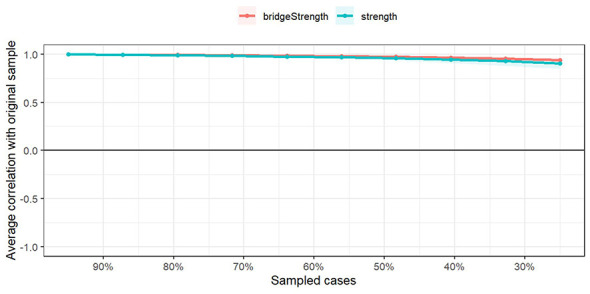
Average correlation with the original sample for different centrality measures across decreasing proportions of the retained sample.

The results showed the stability performance of the bridge Strength and strength indices under conditions of decreasing sample size. When the retained sample size was reduced to 50%, the correlations between these indices and those from the original sample remained above 70%, indicating that these two centrality indices exhibited good stability. These findings suggest that the estimated network showed acceptable stability for strength-related indices in the present sample; however, other centrality indices and the overall network structure should be interpreted cautiously and require further validation (Figure 4).

## Discussion

This study, based on a network analysis approach, explored the associations among burnout, presenteeism, and social support in Chinese pediatric nurses. The results showed that within the emotional exhaustion dimension, D6 (I have become more callous toward work since I took this job) and D5 (I feel burned out from my work) were connected and were associated with E1 (I have become less enthusiastic about my work) and E2 (I doubt the significance of my work) in the depersonalization dimension, indicating that emotional exhaustion plays a role in the symptoms. This finding is consistent with conclusions from related studies (35). However, this study further clarified the mutual influence between emotional exhaustion and depersonalization. Multiple relatively strong blue edges indicated that the relationship between the two is not unidirectional triggering but forms a negative cycle, jointly constituting a relevant mechanism of burnout. The visualization of this process empirically supports Halbesleben's (36) “Conservation of Resources theory,” which proposes that continuous depletion of emotional resources may lead nurses to adopt dehumanized coping responses. Previous studies have shown that social support and burnout affect employees' turnover intention (37). The results also showed that within the presenteeism dimension, “because of health problems, unable to obtain pleasure from work (A3)” occupied an important position, suggesting that loss of professional identity is a key factor leading to decreased work efficiency. At the same time, “I have become less enthusiastic about my work (E1)” and “I have become more callous toward work since I took this job (D6)” in burnout exhibited relatively strong connectivity. This indicates that for pediatric nurses, early intervention for burnout should focus on emotional energy depletion rather than merely increasing manpower input, which is consistent with previous research (38).

Within the social support dimension, C6 (Support from spouse/partner), C9 (Support from siblings), and C11 (Support from other members) showed associations, indicating that family support is the resource. This may also reflect the insufficient medical resources and weak organizational support systems in underdeveloped areas of the surveyed region. However, the social support module was relatively independent in the network, and some nodes (such as C1 and C2) showed relatively low strength, indicating that there remains substantial room for improvement. This finding is consistent with the results of Yang et al., who reported that social support is a negative influencing factor of burnout among healthcare workers (39). Nursing managers should fully recognize the importance of social support for nurses and implement corresponding measures to strengthen it, thereby ensuring the stability of the nursing team (16).

The results of the network analysis also identified relevant nodes for occupational psychological intervention. The findings showed that nodes D6, E1, and C11 had relatively strong influence in the network and played roles in the interrelationships among burnout, presenteeism, and social support. Specifically, D6 (node of emotional exhaustion) should be prioritized to interrupt its vicious cycle with D5 and E1 through emotional counseling, workload adjustment, and related measures. This strategy is supported by the study of Ong, N.Y., et al., which found that mindfulness interventions targeting emotional exhaustion reduced burnout scores among nurses (40). E1 (node of depersonalization) may be addressed by strengthening professional identity and reconstructing a sense of meaning, thereby reducing the risks of emotional exhaustion and depersonalization. For example, implementing “narrative nursing” workshops can help nurses rediscover their professional value (41). C11 (node of social support) should focus on optimizing access to family support and supplementing organizational and colleague support to build a multidimensional protective network. The acceptable stability of strength-related indices and the relatively narrow confidence intervals for several edges provide methodological support for the exploratory interpretation of the main findings. Nevertheless, these results should not be regarded as definitive evidence of a fully stable network structure, and external validation is still required.

This study explored the associations among occupational burnout, presenteeism, and social support in pediatric nurses through network analysis, with the aim of identifying features and items that may play connecting roles among the three constructs and providing a basis for more targeted interventions. Previous studies have shown that social support is not only closely related to the level of occupational burnout among nurses, but also affects their turnover intention and occupational adaptation process (42, 43); at the same time, job stability, perceived stress, and physical fatigue have also been regarded as factors related to the occurrence of burnout (44). However, current burnout interventions commonly used in the field of nursing management mainly involve self-care activities, mindfulness practices, or general psychological support. Although these approaches can alleviate stress responses to some extent, they often fail to fully address the individualized needs of individuals or specific groups (45, 46). In contrast, organizational-level interventions place greater emphasis on the work context itself and seek to strengthen nurses' occupational support systems by reasonably controlling workload, optimizing job content, improving the team collaboration climate, and establishing peer support networks (47, 48). Meanwhile, the regular provision of psychological counseling, emotion management, and stress-coping training may also help enhance nurses' emotional regulation and resource utilization abilities, reduce the risk of occupational burnout, and thereby promote the stability and professional development of nursing teams (49).

## Practical implications

Based on the cross-sectional findings of this study and evidence from previous research, we propose several reference recommendations for future interventions targeting presenteeism and burnout among pediatric nurses.

First, for burnout-related nodes such as reduced interest in work and loss of sense of value, hospitals may regularly organize occupational mental health lectures and group psychological counseling sessions to help nurses alleviate negative emotions, or strengthen professional value identification through recognition mechanisms.

Second, based on the role of sources of comfort and concern when encountering emergencies, a support team composed of senior nurses and departmental leaders may be established to provide immediate emotional counseling and work assistance to nurses facing urgent or critical situations.

Third, hospitals may promote the monitoring of presenteeism through regular work efficiency assessments and questionnaires evaluating nurses' physical and psychological conditions, so as to identify and intervene in relevant groups.

## Limitations

This study has several limitations.

First, this study was a cross-sectional survey, and occupational burnout, presenteeism, and social support were all measured at the same time point. Therefore, the network obtained in this study only reflects the association structure among items, and the results can only be used to identify relative relationships in the current sample; they cannot be used to infer the temporal order, causal direction, or pathways among variables. Accordingly, the conclusions of this study should be interpreted as descriptive findings, which still require further expanded investigations or validation through longitudinal studies.

Second, all data in this study were derived from self-reported scales. Although these scales demonstrated good reliability and validity, they may be affected by social desirability bias and common method bias. In addition, factors such as workload, staffing pressure, emotional labor, work–family conflict, or general distress may constitute influencing factors or pathways underlying part of the observed network structure. Future studies should expand the scope of investigation and further analyze the possible underlying causes in order to improve the validity and explanatory power of the findings.

Third, the sample of this study was drawn from pediatric nurses in Chongqing. Although some network indices in this study showed acceptable stability in the current sample, the relevant findings should still be understood as exploratory and sample-specific. Medical resources, organizational support environments, and cultural backgrounds in different regions may affect the extrapolation of the network structure and key nodes. Therefore, future studies should adopt longitudinal and multicenter designs to further validate the stability of this network pattern across different investigations. In addition, pilot interventions may be conducted for relatively stable and interpretable nodes, and the impact of burnout and presenteeism on nursing care quality indicators should be evaluated.

## Conclusion

This study explored the interactive structure among burnout, presenteeism, and social support in Chinese pediatric nurses based on network analysis. The results showed that presenteeism was associated with burnout, and social support was also associated with both. Network analysis identified emotional exhaustion and social support as nodes influencing nurses' occupational health. The network model showed acceptable stability for selected strength-related indices in the current sample, providing preliminary support for the exploratory findings, but the stability of the overall network structure and specific nodes requires further validation. The results suggest that targeted intervention strategies can be developed based on relevant nodes. Through emotional counseling, reconstruction of professional identity, and establishment of a multidimensional social support system, presenteeism and burnout among pediatric nurses may be alleviated, thereby improving occupational health and the quality of nursing services.

## Data Availability

The original contributions presented in the study are included in the article/Supplementary material, further inquiries can be directed to the corresponding author.
