# Supplementary material for: Network analysis of the relationships among burnout, presenteeism, and social support in Chinese pediatric nurses
Source: Front Public Health. 2026 Jun 25;14:1835612. doi: 10.3389/fpubh.2026.1835612 (PMC13346181; doi:10.3389/fpubh.2026.1835612)
Supplement: Supplementary file 1 [file Table_1.docx]

Supplementary table 1. CS coefficients from global network invariance tests

|  | A1 | A2 | A3 | A4 | B1 | B2 | C1 | C2 | C3 | C4 | C5 | C6 | C7 | C8 | C9 | C10 | C11 | C12 | C13 | C14 | D1 | D2 | D3 | D4 | D5 | D6 | E1 | E2 | E3 | F1 | F2 | F3 | F4 | F5 | F6 |
| --- | --- | --- | --- | --- | --- | --- | --- | --- | --- | --- | --- | --- | --- | --- | --- | --- | --- | --- | --- | --- | --- | --- | --- | --- | --- | --- | --- | --- | --- | --- | --- | --- | --- | --- | --- |
| A1 | 0 | 0.329 | 0.278 | 0.081 | 0.108 | 0 | 0 | 0 | 0 | 0 | -0.031 | 0 | -0.005 | 0 | 0 | 0 | -0.001 | -0.017 | 0 | -0.021 | 0.044 | 0.041 | 0 | 0.028 | 0.018 | 0 | 0 | 0 | 0.014 | 0 | 0 | 0 | 0 | 0.013 | 0 |
| A2 | 0.329 | 0 | 0.278 | 0.406 | 0.004 | 0 | 0 | 0 | 0 | 0 | 0 | 0 | 0 | 0 | 0 | 0 | 0 | 0 | 0 | -0.001 | 0 | 0 | 0 | 0 | 0 | 0 | 0 | 0 | 0.003 | 0 | 0 | 0 | 0 | 0 | 0 |
| A3 | 0.278 | 0.278 | 0 | 0.282 | 0.013 | 0 | 0 | 0 | -0.046 | 0 | -0.012 | 0 | -0.028 | 0 | 0 | -0.009 | 0 | 0 | 0 | -0.025 | 0 | 0 | 0.014 | 0.013 | 0.018 | 0.019 | 0.019 | 0.028 | 0 | 0 | 0 | 0 | 0.029 | 0 | 0 |
| A4 | 0.081 | 0.406 | 0.282 | 0 | 0.023 | 0 | 0 | 0 | 0 | 0 | 0 | 0 | 0 | 0 | 0 | 0 | 0 | 0 | 0 | 0 | 0 | 0 | 0 | 0 | 0 | 0.009 | 0.022 | 0 | 0.005 | 0 | 0 | 0 | 0 | 0 | 0.015 |
| B1 | 0.108 | 0.004 | 0.013 | 0.023 | 0 | 0.721 | 0 | 0 | 0.002 | 0.001 | 0 | 0 | 0 | 0 | 0 | 0 | 0 | 0 | 0 | 0 | 0 | 0 | 0 | 0 | 0 | 0 | 0 | 0 | 0 | 0 | -0.014 | 0 | 0 | 0 | 0 |
| B2 | 0 | 0 | 0 | 0 | 0.721 | 0 | 0 | 0.012 | 0 | 0 | 0 | 0 | 0 | 0.019 | 0.025 | 0.017 | 0 | 0 | 0.012 | 0.007 | 0 | 0 | -0.019 | 0 | 0 | 0 | -0.009 | -0.01 | 0 | 0 | -0.006 | 0 | 0 | -0.02 | -0.012 |
| C1 | 0 | 0 | 0 | 0 | 0 | 0 | 0 | 0.064 | 0.006 | 0.033 | 0 | 0.198 | 0 | 0.102 | 0 | 0 | 0 | 0 | 0.015 | 0 | 0 | 0 | 0 | 0 | 0 | 0 | 0 | 0 | 0 | 0 | 0 | -0.019 | 0 | 0 | 0 |
| C2 | 0 | 0 | 0 | 0 | 0 | 0.012 | 0.064 | 0 | 0.161 | 0 | 0.029 | 0 | 0 | 0.029 | 0.049 | 0.005 | 0 | 0.072 | 0 | 0.081 | 0 | 0 | -0.053 | 0 | 0 | 0 | 0 | 0 | 0 | 0.02 | 0 | 0 | 0 | 0 | 0.026 |
| C3 | 0 | 0 | -0.046 | 0 | 0.002 | 0 | 0.006 | 0.161 | 0 | 0.03 | 0.101 | 0 | 0.066 | 0 | 0.012 | 0 | 0.102 | 0.001 | 0.059 | 0.086 | -0.048 | -0.014 | 0 | 0 | 0 | 0 | 0 | -0.001 | 0 | 0 | 0 | 0 | -0.015 | -0.007 | -0.019 |
| C4 | 0 | 0 | 0 | 0 | 0.001 | 0 | 0.033 | 0 | 0.03 | 0 | 0.089 | 0.025 | 0.009 | 0 | 0 | 0 | 0.491 | 0.023 | 0.033 | 0 | 0 | 0 | 0 | 0 | -0.02 | 0 | 0 | 0 | 0 | 0 | -0.001 | 0 | 0 | 0 | 0 |
| C5 | -0.031 | 0 | -0.012 | 0 | 0 | 0 | 0 | 0.029 | 0.101 | 0.089 | 0 | 0 | 0 | 0 | 0 | 0.07 | 0.085 | 0.076 | 0.086 | 0.014 | 0 | 0 | 0 | 0 | 0 | 0 | 0 | 0 | 0 | 0 | 0 | 0 | -0.01 | 0 | 0 |
| C6 | 0 | 0 | 0 | 0 | 0 | 0 | 0.198 | 0 | 0 | 0.025 | 0 | 0 | 0.184 | 0.325 | 0.016 | 0.029 | 0.117 | 0 | 0 | 0 | 0 | 0 | 0 | 0 | 0 | 0 | 0 | 0 | 0 | 0 | 0 | 0 | 0 | 0 | 0 |
| C7 | -0.005 | 0 | -0.028 | 0 | 0 | 0 | 0 | 0 | 0.066 | 0.009 | 0 | 0.184 | 0 | 0.135 | 0.197 | 0.006 | 0.012 | 0 | 0.122 | 0 | 0 | 0 | 0 | 0 | 0 | 0 | 0 | 0 | 0 | 0 | 0 | 0 | 0 | 0 | -0.005 |
| C8 | 0 | 0 | 0 | 0 | 0 | 0.019 | 0.102 | 0.029 | 0 | 0 | 0 | 0.325 | 0.135 | 0 | 0.107 | 0.298 | 0 | 0 | 0 | 0 | 0 | 0 | -0.016 | 0 | 0 | 0 | 0 | 0 | 0 | 0 | 0 | 0 | 0 | -0.01 | -0.002 |
| C9 | 0 | 0 | 0 | 0 | 0 | 0.025 | 0 | 0.049 | 0.012 | 0 | 0 | 0.016 | 0.197 | 0.107 | 0 | 0.422 | 0.016 | 0 | 0 | 0 | 0 | 0 | 0 | 0 | 0 | 0 | 0 | 0 | 0 | 0 | 0 | 0 | 0 | 0 | 0 |
| C10 | 0 | 0 | -0.009 | 0 | 0 | 0.017 | 0 | 0.005 | 0 | 0 | 0.07 | 0.029 | 0.006 | 0.298 | 0.422 | 0 | 0.021 | 0.001 | 0.057 | 0.032 | 0 | 0 | 0 | 0 | 0 | 0 | 0 | 0 | 0 | 0 | 0 | 0 | 0 | -0.008 | 0 |
| C11 | -0.001 | 0 | 0 | 0 | 0 | 0 | 0 | 0 | 0.102 | 0.491 | 0.085 | 0.117 | 0.012 | 0 | 0.016 | 0.021 | 0 | 0.079 | 0.065 | 0.071 | -0.006 | 0 | -0.018 | 0 | 0 | 0 | 0 | -0.003 | -0.016 | 0 | -0.005 | 0 | -0.015 | 0 | 0 |
| C12 | -0.017 | 0 | 0 | 0 | 0 | 0 | 0 | 0.072 | 0.001 | 0.023 | 0.076 | 0 | 0 | 0 | 0 | 0.001 | 0.079 | 0 | 0.263 | 0.104 | 0 | 0 | -0.001 | -0.006 | -0.004 | -0.003 | 0 | 0 | -0.003 | 0 | 0 | -0.002 | -0.035 | 0 | -0.005 |
| C13 | 0 | 0 | 0 | 0 | 0 | 0.012 | 0.015 | 0 | 0.059 | 0.033 | 0.086 | 0 | 0.122 | 0 | 0 | 0.057 | 0.065 | 0.263 | 0 | 0.113 | 0 | 0 | 0 | 0 | 0 | 0 | 0 | 0 | -0.023 | 0.009 | 0 | 0 | 0 | 0 | 0 |
| C14 | -0.021 | -0.001 | -0.025 | 0 | 0 | 0.007 | 0 | 0.081 | 0.086 | 0 | 0.014 | 0 | 0 | 0 | 0 | 0.032 | 0.071 | 0.104 | 0.113 | 0 | -0.032 | 0 | 0 | 0 | 0 | -0.02 | -0.012 | 0 | 0 | 0 | -0.029 | 0 | 0 | -0.045 | 0 |
| D1 | 0.044 | 0 | 0 | 0 | 0 | 0 | 0 | 0 | -0.048 | 0 | 0 | 0 | 0 | 0 | 0 | 0 | -0.006 | 0 | 0 | -0.032 | 0 | 0.434 | 0.182 | 0.113 | 0.139 | 0.001 | 0.016 | 0 | 0 | -0.05 | 0 | 0 | 0 | 0 | 0 |
| D2 | 0.041 | 0 | 0 | 0 | 0 | 0 | 0 | 0 | -0.014 | 0 | 0 | 0 | 0 | 0 | 0 | 0 | 0 | 0 | 0 | 0 | 0.434 | 0 | 0.154 | 0.14 | 0.057 | 0 | 0 | 0 | 0 | -0.032 | 0 | 0 | 0 | 0 | 0 |
| D3 | 0 | 0 | 0.014 | 0 | 0 | -0.019 | 0 | -0.053 | 0 | 0 | 0 | 0 | 0 | -0.016 | 0 | 0 | -0.018 | -0.001 | 0 | 0 | 0.182 | 0.154 | 0 | 0.365 | 0.04 | 0.087 | 0.113 | 0.001 | 0 | 0 | 0 | 0 | 0 | 0 | 0 |
| D4 | 0.028 | 0 | 0.013 | 0 | 0 | 0 | 0 | 0 | 0 | 0 | 0 | 0 | 0 | 0 | 0 | 0 | 0 | -0.006 | 0 | 0 | 0.113 | 0.14 | 0.365 | 0 | 0.25 | 0.031 | 0 | 0.05 | 0.06 | 0 | -0.014 | 0 | 0 | 0 | 0 |
| D5 | 0.018 | 0 | 0.018 | 0 | 0 | 0 | 0 | 0 | 0 | -0.02 | 0 | 0 | 0 | 0 | 0 | 0 | 0 | -0.004 | 0 | 0 | 0.139 | 0.057 | 0.04 | 0.25 | 0 | 0.268 | 0.068 | 0.061 | 0.045 | 0 | 0 | 0 | 0 | 0 | 0 |
| D6 | 0 | 0 | 0.019 | 0.009 | 0 | 0 | 0 | 0 | 0 | 0 | 0 | 0 | 0 | 0 | 0 | 0 | 0 | -0.003 | 0 | -0.02 | 0.001 | 0 | 0.087 | 0.031 | 0.268 | 0 | 0.307 | 0.222 | 0.123 | 0 | 0 | 0 | 0.024 | 0 | 0 |
| E1 | 0 | 0 | 0.019 | 0.022 | 0 | -0.009 | 0 | 0 | 0 | 0 | 0 | 0 | 0 | 0 | 0 | 0 | 0 | 0 | 0 | -0.012 | 0.016 | 0 | 0.113 | 0 | 0.068 | 0.307 | 0 | 0.438 | 0.152 | -0.002 | 0 | 0 | 0.016 | 0 | 0 |
| E2 | 0 | 0 | 0.028 | 0 | 0 | -0.01 | 0 | 0 | -0.001 | 0 | 0 | 0 | 0 | 0 | 0 | 0 | -0.003 | 0 | 0 | 0 | 0 | 0 | 0.001 | 0.05 | 0.061 | 0.222 | 0.438 | 0 | 0.167 | 0 | 0 | 0 | 0.016 | 0 | 0 |
| E3 | 0.014 | 0.003 | 0 | 0.005 | 0 | 0 | 0 | 0 | 0 | 0 | 0 | 0 | 0 | 0 | 0 | 0 | -0.016 | -0.003 | -0.023 | 0 | 0 | 0 | 0 | 0.06 | 0.045 | 0.123 | 0.152 | 0.167 | 0 | -0.052 | 0 | 0 | 0.029 | 0 | 0 |
| F1 | 0 | 0 | 0 | 0 | 0 | 0 | 0 | 0.02 | 0 | 0 | 0 | 0 | 0 | 0 | 0 | 0 | 0 | 0 | 0.009 | 0 | -0.05 | -0.032 | 0 | 0 | 0 | 0 | -0.002 | 0 | -0.052 | 0 | 0.263 | 0.341 | 0.068 | 0 | 0.093 |
| F2 | 0 | 0 | 0 | 0 | -0.014 | -0.006 | 0 | 0 | 0 | -0.001 | 0 | 0 | 0 | 0 | 0 | 0 | -0.005 | 0 | 0 | -0.029 | 0 | 0 | 0 | -0.014 | 0 | 0 | 0 | 0 | 0 | 0.263 | 0 | 0.383 | 0 | 0.186 | 0 |
| F3 | 0 | 0 | 0 | 0 | 0 | 0 | -0.019 | 0 | 0 | 0 | 0 | 0 | 0 | 0 | 0 | 0 | 0 | -0.002 | 0 | 0 | 0 | 0 | 0 | 0 | 0 | 0 | 0 | 0 | 0 | 0.341 | 0.383 | 0 | 0.064 | 0 | 0.16 |
| F4 | 0 | 0 | 0.029 | 0 | 0 | 0 | 0 | 0 | -0.015 | 0 | -0.01 | 0 | 0 | 0 | 0 | 0 | -0.015 | -0.035 | 0 | 0 | 0 | 0 | 0 | 0 | 0 | 0.024 | 0.016 | 0.016 | 0.029 | 0.068 | 0 | 0.064 | 0 | 0.449 | 0.316 |
| F5 | 0.013 | 0 | 0 | 0 | 0 | -0.02 | 0 | 0 | -0.007 | 0 | 0 | 0 | 0 | -0.01 | 0 | -0.008 | 0 | 0 | 0 | -0.045 | 0 | 0 | 0 | 0 | 0 | 0 | 0 | 0 | 0 | 0 | 0.186 | 0 | 0.449 | 0 | 0.371 |
| F6 | 0 | 0 | 0 | 0.015 | 0 | -0.012 | 0 | 0.026 | -0.019 | 0 | 0 | 0 | -0.005 | -0.002 | 0 | 0 | 0 | -0.005 | 0 | 0 | 0 | 0 | 0 | 0 | 0 | 0 | 0 | 0 | 0 | 0.093 | 0 | 0.16 | 0.316 | 0.371 | 0 |

Note: CS: correlation stability, CS = correlation stability coefficient (range: 0–1). CS > .50 indicates strong stability; CS > .25 indicates acceptable stability. All coefficients were obtained via case-dropping bootstrap using the bootnet package.
